# Silencing of *PCK1* mitigates the proliferation and migration of vascular smooth muscle cells and vascular intimal hyperplasia by suppressing STAT3/DRP1-mediated mitochondrial fission: PCK1/STAT3/DRP1 in VSMC proliferation and migration

**DOI:** 10.3724/abbs.2024154

**Published:** 2024-09-11

**Authors:** Li Zhang, Yingmei Chen, Quanrong Pan, Shizheng Fang, Zhongjian Zhang, Jia Wang, Yongjian Yang, Dachun Yang, Xiongshan Sun

**Affiliations:** 1 Department of Cardiology the General Hospital of Western Theater Command Chengdu 610083 China; 2 Department of General Practice the General Hospital of Western Theater Command Chengdu 610083 China; 3 Department of Critical Care Medicine the General Hospital of Western Theater Command Chengdu 610083 China

**Keywords:** phosphoenolpyruvate carboxyl kinase 1, vascular smooth muscle cells, mitochondrial fission, neointimal hyperplasia, dynamin-related protein 1

## Abstract

The pathological proliferation and migration of vascular smooth muscle cells (VSMCs) are key processes during vascular neointimal hyperplasia (NIH) and restenosis. Phosphoenolpyruvate carboxy kinase 1 (PCK1) is closely related to a variety of malignant proliferative diseases. However, the role of PCK1 in VSMCs has rarely been investigated. This study aims to examine the role of PCK1 in the proliferation and migration of VSMCs and vascular NIH after injury. *In vivo*, extensive NIH and increased expression of PCK1 within the neointima are observed in injured arteries. Interestingly, the administration of adeno-associated virus-9 (AAV-9) carrying *Pck1* short hairpin RNA (sh *Pck1*) significantly attenuates NIH and stenosis of the vascular lumen. *In vitro*, *Pck1* small interfering RNA (si *Pck1*)-induced *PCK1* silencing inhibits VSMC proliferation and migration. Additionally, silencing of *PCK1* leads to reduced expression of dynamin-related protein 1 (DRP1) and attenuated mitochondrial fission. Lentivirus-mediated DRP1 overexpression markedly reverses the inhibitory effects of *PCK1* silencing on VSMC proliferation, migration, and mitochondrial fission. Finally, PCK1 inhibition attenuates the phosphorylation of signal transducer and activator of transcription 3 (STAT3). Activation of STAT3 abolishes the suppressive effects of *PCK1* silencing on DRP1 expression, mitochondrial fission, proliferation, and migration in VSMCs. In conclusion, PCK1 inhibition attenuates the mitochondrial fission, proliferation, and migration of VSMCs by inhibiting the STAT3/DRP1 axis, thereby suppressing vascular NIH and restenosis.

## Introduction

Cardiovascular diseases (CVDs) are the leading cause of death worldwide [1]. As one of the most important treatment methods, percutaneous coronary intervention (PCI) significantly reduces the mortality of coronary heart disease (CHD) [2]. However, mechanical injury initiates a series of pathological processes, causing the development of neointimal hyperplasia (NIH) and ultimately leading to vascular restenosis [3]. The main pathophysiological processes of NIH include endothelial cell damage and dysfunction, inflammatory cell infiltration, the subsequent migration and proliferation of VSMCs and vascular adventitial fibroblasts, and the accumulation of the extracellular matrix (ECM) [ 4– 6]. The abnormal proliferation and migration of VSMCs, the major component of vessels, is one of the most critical pathophysiological mechanisms during NIH [7]. Drug-eluting stents (DESs) that target cell proliferation significantly reduce the rates of vascular restenosis and revascularization. However, its incidence remains as high as 4%–8% [8]. DES not only inhibits cell proliferation but also inhibits vascular re-endothelialization, resulting in a longer duration of DES-related NIH than of bare metal stent (BMS)-related NIH [9]. Therefore, identifying new therapeutic targets and developing new drug stents or balloons are crucial for preventing restenosis.

PCK1 was originally identified as a key rate-limiting enzyme of gluconeogenesis. Furthermore, PCK1 is highly enriched in the liver, kidney, intestine, and adipose tissue and regulates the production of glucose, fatty acid re-esterification and citric acid cyclic anions [ 10– 12]. It is closely related to the development of diabetes, obesity, cardiovascular disease, and tumors [ 12, 13]. In addition, PCK1 is involved in the biosynthesis of serine, an amino acid that is the major substrate for one-carbon metabolism; is vital for nucleotide synthesis, methylation, and antioxidation; and is associated with the migration and proliferation of tumor cells [ 14, 15]. Moreover, PCK1 also promotes protein kinase activity to activate sterol regulatory element binding proteins (SREBPs), resulting in the transcription of lipogenesis genes and promoting the rapid proliferation of tumor cells [16]. Therefore, PCK1 can promote the development of various malignant proliferative diseases, such as non-small cell lung cancer, breast cancer, colon cancer, and hepatocellular carcinoma [ 11, 17, 18]. Moreover, PCK1 is involved in vascular endothelial function. In a recent study, PCK1 shRNA was found to inhibit the proliferation and migration and decrease the number of tube-like structures and average sprouting length in human umbilical vein endothelial cells. These characteristics indicate that silencing of *PCK1* results in antiangiogenic activity through mediating AKT inactivation and subsequent Gα _i3_ downregulation [19]. Additionally, *in vivo* knockdown of *PCK1* disrupts retinal angiogenesis in mice. However, the biological function of PCK1 in the proliferation and migration of VSMCs has rarely been investigated, and the underlying mechanisms have rarely been explored.

Mitochondria are highly dynamic organelles that produce the majority of the energy needed for normal cellular function [20]. Mitochondria continually undergo fission and fusion to maintain their stable morphology and function, which is referred to as mitochondrial dynamics [21]. Mitochondrial fission refers to the process in which one mitochondrion divides into two mitochondria [ 22, 23]. Mitochondrial fission is involved in the inheritance and allocation of organelles during cell proliferation, the release of cytochrome C during cell apoptosis, and the removal of damaged mitochondria by autophagy [ 24– 26]. Studies have revealed that mitochondrial fission is also involved in the proliferation and migration of VSMCs and NIH [ 27, 28]. Mitochondrial fission is regulated by various proteins, such as mitochondrial dynamics proteins 49 (MID49) and 51 (MID51), mitochondrial fission factor (MFF), mitochondrial fission 1 protein (FIS1), and mitochondrial DRP1 [ 22, 28, 29]. Among these proteins, DRP1 plays a key role in the regulation of mitochondrial fission. MFF, MID49, and MID51 regulate mitochondrial fission by recruiting the activated DRP1 adapter to the outer mitochondrial membrane (OMM) [30]. However, whether DRP1-mediated mitochondrial fission is involved in the regulatory effect of PCK1 on VSMCs remains unclear.

In the present study, we demonstrated that PCK1 was upregulated in injured arteries. Silencing of *PCK1* inhibited the proliferation and migration of VSMCs and injury-induced NIH. Mechanistically, attenuated mitochondrial fission was observed in VSMCs after PCK1 expression was downregulated. Additionally, we found that DRP1 expression was downregulated in VSMCs after *PCK1* was silenced. Exogenous expression of DRP1 abolished the inhibitory effects of *PCK1* silencing on the mitochondrial fission, proliferation, and migration of VSMCs. Our study may provide a novel target for preventing vascular restenosis after PCI.

## Materials and Methods

### Animals

All experiments involving animals were approved by the Institutional Animal Care and Use Committee and the Ethics Committee of the General Hospital of Western Theater Command (2023EC5-ky002) and were performed in accordance with the ARRIVE (Animal Research: Reporting of *In Vivo* Experiments) guidelines. Male C57BL/6J mice (7–8 weeks old) were purchased from Chengdu YaoKang Biological Technology Co., Ltd. (Chengdu, China). The mice were housed in a clean animal facility with a 12/12-h light/dark cycle at 22–25°C and had free access to water and food. The experimental animals were randomly divided into four groups: the sham group ( *n*  = 23), injury group ( *n*  = 28), sham + sh *Pck1* group ( *n*  = 25), and injury+sh *Pck1* group ( *n*  = 27). After one week of adaptive feeding, the injury model was established as described previously [31]. First, the mice were anaesthetized by an intraperitoneal injection of pentobarbital sodium (30 mg/kg), and a small midline incision was made on the neck after depilation and disinfection of the skin. Next, the left common, internal, and external carotid arteries were exposed through blunt dissection. The left external carotid artery was ligated. The common and internal carotid arteries were briefly clamped with vascular clamps. Then, a thick guidewire was inserted into the common carotid artery and rotated repeatedly to damage the vascular endothelium. Finally, the surgical incision was sutured after restoring the blood supply by removing the clamps. The mice in the sham operation group were not injured by the guidewire. The other procedures were the same as above. Four weeks after surgery, the mice were euthanized with pentobarbital sodium (100 mg/kg). The left common carotid arteries were isolated and collected for subsequent experiments.

### Adeno-associated virus serotype 9 (AAV-9) infection *in vivo*

We constructed an AAV-9 vector carrying *Pck1* short hairpin RNA (sh *Pck1*) (sequence: 5′-CCAAGATCTTCCATGTCAA-3′; Genechem, Shanghai, China) according to the manufacturer’s instructions. AAV-9 was immediately injected into the injured left common carotid artery after the proximal and distal ends were clamped. After incubation for 30 min, the blood supply was restored, and the incision was closed [32].

### Hematoxylin and eosin (H&E) analysis

H&E staining was performed for morphological analysis of the carotid artery as previously reported [33]. The left common carotid artery was isolated at 28 days after surgery and fixed with 4% paraformaldehyde (PFA) after perfusion with phosphate-buffered saline (PBS). The tissues were subsequently dehydrated in ethanol, cleared with xylene, and embedded in paraffin. Next, the tissues were cut into 4-μm pieces, dewaxed with xylene, rehydrated with gradient alcohol, and stained with hematoxylin and eosin. After washing, the tissue sections were dehydrated and sealed. Finally, images were captured via a general light microscope (Nikon, Tokyo, Japan) and analyzed by a slide scanner (Olympus, Tokyo, Japan).

### Immunofluorescence analysis

Immunofluorescence analysis was performed as previously reported [16]. For tissue staining, paraffin sections (4 μm) of the carotid artery were dewaxed, rehydrated, and subjected to heat-mediated antigen retrieval. Next, the sections were blocked in 1% bovine serum albumin (BSA; BioFoxx, Munich, Germany) for 30 min at room temperature and then incubated with primary antibodies against alpha smooth muscle actin (α-SMA, 1:200; Biosharp, Shanghai, China), PCK1 (1:200; Abcam, Cambridge, UK), or Ki-67 (1:500; Bioss, Beijing, China) overnight at 4°C. The next day, the sections were stained with Alexa Fluor 488-conjugated goat anti-rabbit and Alexa Fluor 594-conjugated goat anti-mouse secondary antibodies (1:2000; Invitrogen, Carlsbad, USA) in the dark for 1 h and then stained with DAPI (10 mg/mL; Biosharp) for 5 min at room temperature. For cellular staining, after being washed with PBS, cultured VSMCs were fixed with 4% PFA for 30 min, treated with 0.1% Triton X-100 for 5 min, and blocked in 1% blocking solution for 30 min. Then, the VSMCs were incubated with primary antibodies against Ki-67 or PCK1 overnight at 4°C. After washing, the VSMCs were incubated in the dark with Alexa Fluor 488-conjugated goat anti-rabbit and Alexa Fluor 594-conjugated goat anti-mouse secondary antibodies (1:1000) for 1 h and then stained with DAPI (10 mg/mL) for 5 min at room temperature. For live-cell staining, the VSMCs were stained in the dark with 200 nM MitoTracker Green (Beyotime Biotechnology, Shanghai, China) in complete medium for 30 min and then with 10 μL/mL Hoechst 33342 Staining Solution for 10 min at 37°C (Beyotime Biotechnology). Immunofluorescence microscopy images were obtained with a confocal microscope (Nikon).

### VSMC culture and treatments

VSMCs were isolated from the thoracic aortas of male C57BL/6J mice aged 8–10 weeks as previously described [34]. The VSMCs were cultured in complete medium containing high-glucose Dulbecco’s modified Eagle’s medium (DMEM; HyClone, Carlsbad, USA), 10% fetal bovine serum (FBS; Gibco, Grand Island, USA), and 1% penicillin-streptomycin (HyClone). The VSMCs were cultured in an incubator at 37°C with 5% CO _2_ and 95% relative humidity. Primary VSMCs were divided into two groups: the scramble siRNA group and the si *Pck1* group. For colivelin treatment, VSMCs were incubated with colivelin (10 μM) for 24 h [35].

### Western blot analysis

The cells were lysed in ice-cold RIPA lysis buffer (Beyotime Biotechnology). Proteins were obtained from the lysates via high-speed centrifugation at 4°C, separated by 10% SDS-PAGE, and then transferred onto polyvinylidene fluoride membranes (Millipore, Billerica, USA). The membranes were subsequently blocked with 5% skim milk diluted with Tris-buffered saline-Tween-20 (TBST) for 1 h at room temperature and then incubated overnight with primary antibodies against PCK1 (1:1000; Abcam), STAT3 (1:1000; Abcam), DRP1 (1:1000; Abcam) and GAPDH (1:10000; Proteintech Group, Wuhan, China) diluted in 5% skim milk at 4°C. The next day, the membranes were washed with TBST for 1 h and incubated with an HRP-conjugated secondary antibody (1:10000; Proteintech Group) for 1 h at room temperature. The development step was conducted with a brand chemiluminescence imager (Azure 300; Azure Biosystems, Dublin, USA). The quantitative analysis was conducted with ImageJ software (NIH, Bethesda, USA).

### Lentivirus transfection and stable cell line generation

According to the manufacturer’s instructions, we constructed a recombinant lentivirus encoding the *Drp1* (LV- *Drp1*; Obio Technology, Shanghai, China) vector and a negative control (LV) vector. VSMCs were first seeded into 24-well plates at a density of 1 × 10 ^4^ cells per well for 24 h. The next day, the VSMCs were transfected with LV- *Drp1* at multiplicities of infection (MOIs) of 0, 10, 20, 50, 100, or 200. The medium was replaced 16 h after transfection. The transfection efficiency and the optimal MOI were determined by fluorescence microscopy 72 h after transfection. The medium was subsequently removed and replaced by complete medium containing 5 μg/mL puromycin to screen stably transfected cells and establish a stable cell line. Finally, the transfection efficiency was measured by western blot analysis.

### siRNA transfection

VSMCs were first seeded in 6-well plates and cultured to 30% confluence. According to the manufacturer’s instructions, VSMCs were then transfected with 100 nM target-specific si *Pck1* (RiboBio, Guangzhou, China) or scramble siRNA by using Lipofectamine ^TM^ 3000 reagent (Invitrogen). After 8 h of transfection, the VSMCs were washed three times with PBS and cultured in fresh serum-containing medium for another 48 h. Ultimately, the transfection efficiency was measured by western blot analysis. The target sequence of si *Pck1* was 5′-CCGCAAGCTGAAGAAATAT-3′. The target sequence of scramble siRNA was 5′-UGGUUUACAUGUCGACUAA-3′.

### Cell proliferation assay

The proliferation of VSMCs was analyzed by the Cell Counting Kit-8 (CCK-8) assay and Ki-67 immunofluorescence staining as reported previously [ 27, 36]. For the CCK-8 assay, treated VSMCs were seeded in 96-well plates at a density of 1 × 10 ^4^ cells per well and cultured for 24 h and then incubated with CCK-8 reagent (1:10; Bioshap) for 1 h at 37°C. The absorbance was measured at 450 nm by using a microplate reader (Bio-Rad, Hercules, USA). For Ki-67 immunofluorescence staining, treated VSMCs were seeded in 6-well plates at a density of 3 × 10 ^4^ cells per well and cultured for 24 h, and then stained with an anti-Ki-67 antibody. Images were obtained via immunofluorescence microscopy (Nikon) and analyzed with ImageJ software.

### Cell migration assay

The migration of VSMCs was evaluated with a wound healing assay as described previously [27]. VSMCs were first seeded in 6-well plates at a density of 1 × 10 ^5^ cells per well and cultured to 90% confluence. After overnight starvation under serum-free conditions, scratch wounds were generated by using a 200-μL sterile pipette tip. Images were obtained by using a microscope (Nikon) at 0 and 24 h after wounding. The rates of wound closure were estimated by ImageJ software.

### Transmission electron microscopy (TEM)

As described previously [37], VSMCs were seeded in 10-cm plates, cultured to approximately 30% confluence, and then treated as described above. Next, the VSMCs were prefixed with 3% glutaraldehyde, postfixed in 1% osmium tetroxide, dehydrated in a series of acetone solutions, infiltrated with Epox 812, and embedded. The semithin sections were stained with methylene blue, and ultrathin sections were cut with a diamond knife and stained with uranyl acetate and lead citrate. The sections were examined with a JEM-1400-FLASH transmission electron microscope (JEM, Tokyo, Japan).

### Statistical analysis

All statistical data are presented as the mean ± SD. Pairwise comparisons were performed using a two-tailed Student’s *t* test. For three or more groups, one-way analysis of variance (ANOVA) was conducted to compare means that involved one or two factors and determine the significance of the experimental results. *P *​< 0.05 was considered to indicate statistical significance.

## Results

### Silencing of *PCK1* alleviates NIH after vascular injury

To investigate the role of PCK1 in NIH after vascular injury, we first determined the expression level of PCK1 in injured arteries by immunofluorescence staining. The fluorescence intensity of PCK1 was significantly greater in the mice that underwent vascular injury than in the mice that underwent a sham operation ( Figure 1A). To determine the distribution of PCK1 in blood vessels, we performed immunofluorescence colocalization of PCK1 with α-SMA, a VSMC marker, and found that PCK1 expression was increased mainly within the neointima ( Figure 1A). After confirming the silencing of *PCK1* *in vivo* by AAV-9-mediated sh *Pck1* treatment, we found that the NIH area was decreased after vascular injury ( Figure 1A and Supplementary Figure S1). In addition, we observed consistent results in the morphological analysis by HE staining. At 28 days after vascular injury, excessive NIH and vascular lumen stenosis were observed in the injured artery, as reflected by an increased intima/media ratio. However, PCK1 inhibition significantly reduced the intima/media ratio and suppressed NIH and vascular stenosis ( Figure 1B). Next, we performed immunofluorescence staining for Ki-67 to further verify the role of PCK1 in NIH. Vascular injury markedly increased the percentage of Ki-67-positive cells within the neointima, which was blocked by silencing of *PCK1* ( Figure 1C). These results suggest that inhibiting PCK1 can ameliorate vascular injury-induced NIH.

**Figure 1:** Figure 1 Silencing of *PCK1* ameliorates neointimal hyperplasia after vascular injury (A,B) Left common carotid arteries of mice 28 days after injury were stained with PCK1 (red), α-SMA (green), and DAPI (blue). Representative images and corresponding quantification of PCK1 fluorescence intensity are shown (n = 6). Scale bar: 50 μm. (C,D) Representative images of H&E staining of the left common carotid arteries of mice 28 days after injury and the corresponding quantification of the intima/media ratio are shown (n = 6). Scale bar: 50 μm. (E,F) Left common carotid arteries of mice 28 days after injury were stained with Ki-67 (red), α-SMA (green), and DAPI (blue). Representative images and the corresponding percentages of Ki-67-positive cells within the intima are shown (n = 6). Scale bar: 50 μm. **P < 0.01 and ***P < 0.001.

### Silencing of *PCK1* inhibits the proliferation and migration of VSMCs

To further confirm the role of PCK1 in the proliferation and migration of VSMCs *in vitro*, we transfected VSMCs with si *Pck1* to downregulate PCK1 expression and analyzed the transfection efficiency by western blot analysis and immunofluorescence staining. The results revealed that PCK1 expression was successfully downregulated after si *Pck1* transfection ( Figure 2A). Moreover, immunofluorescence analysis revealed that PCK1 was located mainly in the cytoplasm ( Figure 2B). Next, we performed a CCK-8 assay and immunofluorescence staining of Ki-67 to evaluate the effect of *PCK1* silencing on VSMC proliferation. The results showed that silencing of *PCK1* significantly reduced cell viability ( Figure 2C). The percentage of Ki-67-positive VSMCs was also reduced by si *Pck1* ( Figure 2D). Thus, we confirmed that PCK1 inhibition could suppress the proliferation of VSMCs. Moreover, wound healing assay revealed that PCK1 inhibition strongly decreased the wound healing rate ( Figure 2E), indicating that the migration of VSMCs was weakened. These results indicate that downregulating PCK1 expression can inhibit the proliferation and migration of VSMCs.

**Figure 2:** Figure 2 The proliferation and migration of VSMCs are hindered by *PCK1* silencing (A,B) VSMCs were transfected with scramble siRNA or siPck1 for 48 h. Representative western blots and corresponding quantification of PCK1 in VSMCs from different groups are shown (n = 5). (C,D) VSMCs from different groups were stained with PCK1 (red) and DAPI (blue). Representative images and corresponding quantification of PCK1 fluorescence intensity are shown (n = 6). Scale bar: 20 μm. (E) The viability of VSMCs from different groups was measured via CCK-8 assay (n = 6). (F,G) VSMCs from different groups were stained with Ki-67 (red) and DAPI (blue). Representative images and the corresponding percentages of Ki-67-positive VSMCs are shown (n = 6). Scale bar: 20 μm. (H,I) The migration ability of VSMCs from different groups was assessed via wound healing assay. Representative images and corresponding quantification of the wound healing rates are shown (n = 5). Scale bar: 100 μm. **P < 0.01 and ***P < 0.001.

### Inhibition of PCK1 reduces mitochondrial fission in VSMCs

Mounting evidence suggests that mitochondrial fission is a critical step for the proliferation and migration of VSMCs and the progression of NIH [ 38– 41]. Hence, we examined mitochondrial dynamics in VSMCs via TEM and found that PCK1 inhibition led to decreased mitochondrial fragmentation and fission, which was characterized by increases in mitochondrial size and length ( Figure 3A). We subsequently examined the morphology of the mitochondria by MitoTracker staining. The ratio of elongated mitochondria in si *Pck1*-treated VSMCs was significantly greater than that in control VSMCs ( Figure 3B). DRP1 promotes mitochondrial fission and the proliferation and migration of VSMCs, ultimately contributing to NIH [42]. Interestingly, the results of the present study revealed that the protein expression of DRP1 was reduced in si *Pck1*-transfected VSMCs ( Figure 3C). Thus, these results indicate that inhibiting PCK1 may inhibit the proliferation and migration of VSMCs by impairing mitochondrial fission.

**Figure 3:** Figure 3 Silencing of *PCK1* suppresses mitochondrial fission in VSMCs through reducing the expression of DRP1 VSMCs were transfected with scramble siRNA or siPck1 for 48 h. (A,B) Representative TEM images of the above-treated VSMCs and quantification of the mean mitochondrial length are shown (n = 9). Scale bar: 500 nm. (C,D) Above-treated VSMCs were stained with MitoTracker (green) and DAPI (blue). Representative images and the corresponding percentages of fragmented mitochondria are shown (n = 6). Scale bar: 5 μm. (E,F) Representative western blots and corresponding quantification of DRP1 in VSMCs from different groups are shown (n = 5). ***P < 0.001.

### DRP1 overexpression abolishes the inhibitory effects of PCK1 inhibition on the mitochondrial fission, proliferation, and migration of VSMCs

To further validate the role of DRP1 in the PCK1-mediated regulation of mitochondrial fission, a lentivirus carrying LV- *Drp1* was transfected into VSMCs to induce DRP1 overexpression. Western blot analysis revealed that lentivirus-mediated DRP1 overexpression was successfully achieved in VSMCs and reversed the inhibitory effect of *PCK1* silencing on DRP1 expression ( Figure 4A). Next, we analyzed the alterations in mitochondrial dynamics using TEM and MitoTracker staining. The results revealed that the overexpression of DRP1 led to increased mitochondrial fission, manifested by increased mitochondrial fragmentation. Additionally, DRP1 overexpression weakened the inhibitory effect of *PCK1* silencing on mitochondrial fission ( Figure 4B,C). These results indicate that PCK1 inhibition suppresses mitochondrial fission by reducing the expression of DRP1.

**Figure 4:** Figure 4 The inhibitory effect of PCK1 on the mitochondrial fission of VSMCs is attenuated via lentivirus-mediated overexpression of DRP1 VSMCs stably transfected with empty vector or lentivirus carrying Drp1 were incubated with scramble siRNA or siPck1 for 48 h. (A,B) Representative western blots and corresponding quantification of PCK1 and DRP1 in VSMCs from different groups are shown (n = 5). (C,D) Representative TEM images and quantification of the mean mitochondrial length in VSMCs from various groups are shown (n = 9). Scale bar: 500 nm. (E,F) Above-treated VSMCs were stained with MitoTracker (green) and DAPI (blue). Representative images and the corresponding percentages of fragmented mitochondria are shown (n = 6). Scale bar: 5 μm. **P < 0.01 and ***P < 0.001.

We further investigated whether the inhibition of PCK1 represses the proliferation and migration of VSMCs by reducing DRP1 expression. CCK-8 and Ki-67 immunofluorescence assays revealed that DRP1 overexpression promoted VSMC proliferation, as reflected by increases in cell viability and the percentage of Ki-67-positive cells. However, the inhibitory effect of *PCK1* silencing on the proliferation of VSMCs was attenuated after exogenous expression of DRP1 ( Figure 5A,B). The migration of VSMCs was subsequently examined by wound healing assay. The results showed that DRP1 overexpression enhanced VSMC migration, as characterized by an increase in the wound healing rate. After DRP1 was overexpressed, the inhibitory effect of *PCK1* silencing on VSMC migration was abolished ( Figure 5C). These results suggest that silencing of *PCK1* inhibits the mitochondrial fission, proliferation and migration of VSMCs by reducing DRP1 expression.

**Figure 5:** Figure 5 DRP1 overexpression reverses the inhibitory effect of *PCK1* silencing on the proliferation and migration of VSMCs VSMCs stably transfected with empty vector or lentivirus carrying Drp1 were treated with scramble siRNA or siPck1 for 48 h. (A) The viability of VSMCs from different groups was measured via CCK-8 assay (n = 6). (B,C) VSMCs from different groups were stained with Ki-67 (red) and DAPI (blue). Representative images and the corresponding percentages of Ki-67-positive VSMCs are shown (n = 6). Scale bar: 20 μm. (D,E) The migration of VSMCs from different groups was assessed via wound healing assay. Representative images and corresponding quantification of the wound healing rates are shown (n = 5). Scale bar: 100 μm. **P < 0.01 and ***P < 0.001.

### PCK1 inhibition reduces DRP1 expression in VSMCs in a STAT3-dependent manner

We next investigated how *PCK1* silencing affects DRP1 expression in VSMCs. A recent study demonstrated that increased STAT3 phosphorylation could lead to the upregulation of DRP1 expression in hepatocellular carcinoma [43]. However, whether STAT3 participates in the PCK1-mediated regulation of DRP1 in VSMCs is unknown. We demonstrated that silencing of *PCK1* attenuated STAT3 phosphorylation in VSMCs ( Figure 6A). Furthermore, the application of colivelin, an agonist of STAT3 [35], reversed the inhibitory effects of *PCK1* silencing on STAT3 phosphorylation, DRP1 expression, and mitochondrial fission ( Figure 6B,C).

**Figure 6:** Figure 6 Reactivation of STAT3 attenuates the inhibitory effects of *PCK1* silencing on DRP1 expression, mitochondrial fission, proliferation and migration in VSMCs (A,B) VSMCs were treated with scramble siRNA or siPck1 for 48 h. Representative western blots and corresponding quantification of pSTAT3/STAT3 in VSMCs from different groups are shown (n = 5). (C,D) Above-treated VSMCs were simultaneously treated with vehicle or colivelin for 4 h. Representative western blots and corresponding quantification of PCK1, DRP1 and pSTAT3/STAT3 in VSMCs from different groups are shown (n = 5). (E,F) VSMCs from different groups were stained with Ki-67 (red) and DAPI (blue). Representative images and the corresponding percentages of Ki-67-positive VSMCs from different groups are shown (n = 6). Scale bar: 20 μm. (G,H) Representative images and corresponding quantification of the wound healing rates of VSMCs from different groups are shown (n = 5). Scale bar: 100 μm. (I,J) Above-treated VSMCs were stained with MitoTracker (green) and DAPI (blue). Representative images and the corresponding percentages of fragmented mitochondria are shown (n = 6). Scale bar: 5 μm. **P < 0.01 and ***P < 0.001.

We next explored the role of STAT3/DRP1 in the PCK1-mediated regulation of VSMCs. As expected, activation of STAT3 by colivelin obviously increased the percentage of Ki-67-positive VSMCs and the wound healing rate. However, the suppressive effect of *PCK1* silencing on the proliferation and migration of VSMCs was counteracted by the restoration of STAT3 activity ( Figure 6D,E).

## Discussion

Vascular NIH is a pathophysiologic response to vascular injury during PCI [44]. Endothelial denudation caused by mechanical damage, as an initial factor, results in peripheral endothelial cell dysfunction, platelet aggregation, and the release of proinflammatory cytokines and growth factors. These factors then lead to inappropriate migration and uncontrolled proliferation of VSMCs, ultimately causing extensive NIH and vascular restenosis [ 45– 48]. In this study, our results demonstrated that silencing of *PCK1* alleviates injury-induced vascular NIH by suppressing the migration and proliferation of VSMCs. Mechanistically, PCK1 inhibition represses mitochondrial fission by attenuating STAT3 phosphorylation-induced DRP1 upregulation in VSMCs.

PCK1 is recognized as a critical enzyme in gluconeogenesis and plays a critical role in catalyzing the conversion of oxaloacetic acid (OAA) to phosphoenolpyruvate (PEP) [49]. The canonical effects of PCK1 include glucose biosynthesis, glycerol biosynthesis, serine biosynthesis, and amino acid metabolism [12]. In addition, PCK1 can also act as a protein kinase to perform nonmetabolic functions. For example, PCK1 is involved in the proliferation and migration of various tumor cells and vascular endothelial cells through metabolic and nonmetabolic effects [14]. However, the potential role of PCK1 in the proliferation and migration of VSMCs and vascular NIH has rarely been explored. In the present study, we utilized a wire injury mouse model to investigate the pathophysiological process of vascular NIH. We observed significantly increased PCK1 expression and proliferating cells within the neointima, extensive NIH and severe lumen stenosis at 28 days after injury. Interestingly, PCK1 inhibition markedly attenuated vascular NIH and stenosis. VSMCs switch from a static “contraction” phenotype to a highly migratory and proliferative “synthetic” phenotype after vascular injury, which is a critical pathological process in the development of the NIH [50]. Consistent with the *in vivo* results, silencing of *PCK1* *in vitro* with si *Pck1* noticeably suppressed the proliferation and migration of VSMCs. Therefore, PCK1 might be a potent therapeutic target for preventing vascular restenosis after vascular injury.

Mitochondrial dynamics regulate the size, quantity, shape, and distribution of mitochondria [51]. Fission produces short tubular or spherical mitochondria, which promote cell proliferation and migration [23]. Many studies have shown that mitochondrial dynamics are involved in phenotypic transition and energy metabolism in VSMCs and are associated with various cardiovascular diseases, such as pulmonary hypertension, coronary artery disease, and restenosis after coronary intervention [52]. There is evidence that excessive mitochondrial fission causes the proliferation and migration of VSMCs and NIH after vascular injury, which can be reversed by blocking mitochondrial fission [39]. PCK1 has been reported to induce mitochondrial dysfunction in the liver [53]. However, PCK1 is essential for mitochondrial function in renal tubular cells [54]. However, whether PCK1 affects mitochondrial fission in VSMCs remains unknown. We observed that silencing of *PCK1* decreased the number of mitochondrial fragments in VSMCs. Thus, PCK1 inhibition might suppress the proliferation and migration of VSMCs by repressing mitochondrial fission.

DRP1 is a key regulatory protein of mitochondrial fission. Increased expression of DRP1 results in accelerated mitochondrial fission, thus promoting the proliferation and migration of cells [55]. However, whether DRP1 is involved in PCK1-mediated regulation of mitochondrial fission in VSMCs is unclear. Our current study revealed that silencing of *PCK1* reduced the expression level of DRP1 in VSMCs. However, overexpression of DRP1 reversed the inhibitory effects of *PCK1* silencing on the mitochondrial fission, proliferation, and migration of VSMCs. Thus, PCK1-mediated regulation of DRP1 expression may be responsible for the alteration of mitochondrial dynamics in VSMCs. However, how PCK1 affects DRP1 expression remains unknown.

STAT3 is a canonical transcription factor that promotes the proliferation and migration of VSMCs [56]. There is also evidence in hepatocellular carcinoma that increased STAT3 phosphorylation stimulates the expression of DRP1 [43]. However, whether PCK1 affects DRP1 expression in VSMCs in a STAT3-dependent manner remains unknown. In the present study, STAT3 phosphorylation was attenuated by *PCK1* silencing in VSMCs. After the restoration of STAT3 phosphorylation by colivelin, the reduction in DRP1 expression and attenuation of mitochondrial fission, proliferation, and migration in VSMCs induced by si *Pck1* were partly reversed. Therefore, we demonstrated that knockdown of *PCK1* decreases DRP1 expression in VSMCs by attenuating STAT3 phosphorylation.

There are several limitations in our current study. First, whether PCK1 affects STAT3 phosphorylation via direct interaction or indirect regulation is unclear. Moreover, our *in vivo* experiments could not exclude the effect of PCK1 on endothelial function. These concepts need further investigation in the future. In conclusion, inhibition of PCK1 can suppress the migration and proliferation of VSMCs during NIH development by attenuating STAT3 phosphorylation and subsequent DRP1-mediated mitochondrial fission ( Figure 7). Unlike canonical drug-eluting stents that target mTOR or other pathways, PCK1 might be considered a potential target for cardiovascular diseases associated with vascular restenosis on the basis of its unique regulation of the STAT3/DRP1 pathway.

**Figure 7:** Figure 7 Silencing of *PCK1* inhibits the migration and proliferation of VSMCs and vascular neointimal hyperplasia by suppressing STAT3/DRP1-mediated mitochondrial fission PCK1 silencing inhibits STAT3 phosphorylation and subsequent STAT3-mediated DRP1 expression and suppresses mitochondrial fission, proliferation and migration in VSMCs and neointimal hyperplasia. This figure was provided by the Home for Researchers, and was generated by using Figdraw.

## Supporting information

24327upplementary_information

## Supplementary Data

Supplementary Data is available at *Acta Biochimica et Biophysica Sinica* online.
