# Supplementary material for: Silencing of PCK1 mitigates the proliferation and migration of vascular smooth muscle cells and vascular intimal hyperplasia by suppressing STAT3/DRP1-mediated mitochondrial fission : PCK1/STAT3/DRP1 in VSMC proliferation and migration
Source: Acta Biochim Biophys Sin (Shanghai). 2024 Sep 11;57(4):633–45. doi: 10.3724/abbs.2024154 (PMC12040600; doi:10.3724/abbs.2024154)
Supplement: 24327upplementary_information [file 24327upplementary_information.pdf]

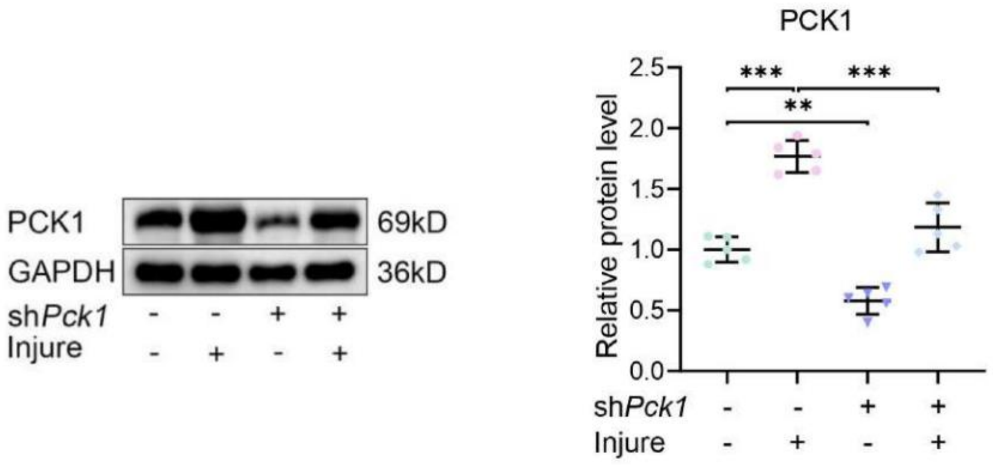

Supplementary Figure S1. PCK1 expression is inhibited after transfection with sh*Pck1*
Representative western blots and corresponding quantification of PCK1 in left common carotid arteries from different groups were shown (*n*=5). ***P*<0.01, ****P*< 0.001.
